# Co-treatments to Boost IDO Activity and Inhibit Production of Downstream Catabolites Induce Durable Suppression of Experimental Autoimmune Encephalomyelitis

**DOI:** 10.3389/fimmu.2020.01256

**Published:** 2020-06-17

**Authors:** Henrique Lemos, Eslam Mohamed, Rong Ou, Caroline McCardle, Xiaozhong Zheng, Kris McGuire, Natalie Z. M. Homer, Damian J. Mole, Lei Huang, Andrew L. Mellor

**Affiliations:** ^1^Immune Metabolism Laboratory, Faculty of Medical Sciences, Translational and Clinical Research Institute, Newcastle University, Newcastle upon Tyne, United Kingdom; ^2^H. Lee Moffitt Cancer Center & Research Institute, Tampa, FL, United States; ^3^Centre for Inflammation Research, University of Edinburgh, Edinburgh, United Kingdom; ^4^Mass Spectrometry Core, Edinburgh Clinical Research Facility, Centre for Cardiovascular Sciences, Queen's Medical Research Institute, Edinburgh, United Kingdom

**Keywords:** autoimmunity, STING, IDO, immunotherapy, EAE

## Abstract

Reinforcing defective tolerogenic processes slows progression of autoimmune (AI) diseases and has potential to promote drug-free disease remission. Previously, we reported that DNA nanoparticles (DNPs) and cyclic dinucleotides (CDNs) slow progression of experimental autoimmune encephalomyelitis (EAE), a mouse model of multiple sclerosis, by activating the Stimulator of Interferon Genes (STING) signaling adaptor to stimulate interferon type 1 (IFN-I) production, which induced dendritic cells to express indoleamine 2,3 dioxygenase (IDO) and acquire immune regulatory phenotypes. Here, we show that therapeutic responses to DNPs depend on DNA sensing via cyclic GAMP synthase (cGAS) and interactions between Programmed Death-1 (PD-1) and PD-1 ligands. To investigate how increased tryptophan (Trp) metabolism by IDO promotes therapeutic responses mice were co-treated at EAE onset with DNPs and drugs that inhibit kynurenine aminotransferase-II (KatII) or 3-hydroxyanthranilic acid dioxygenase (HAAO) activity downstream of IDO in the kynurenine (Kyn) pathway. DNP and KatII or HAAO inhibitor co-treatments suppressed EAE progression more effectively than DNPs, while KatII inhibition had no significant therapeutic benefit and HAAO inhibition attenuated but did not prevent EAE progression. Moreover, therapeutic responses to co-treatments were durable as EAE progression did not resume after co-treatment. Thus, using STING agonists to boost IDO activity and manipulating the Kyn pathway downstream of IDO is an effective strategy to enhance tolerogenic responses that overcome autoimmunity to suppress EAE progression.

## Introduction

Sustained interferon type I (IFN-I) production by activated dendritic cells (DCs) is widely regarded as a common driver of autoimmune (AI) syndromes in humans and mice (1). Under aseptic conditions, some DCs produce IFN-I when they sense damage associated molecular patterns (DAMPs) such as DNA. A critical role for tissue DNA as an autoimmune trigger emerged in mice lacking DNA catabolizing enzymes, which succumbed to spontaneous, lethal autoimmunity due to sustained cytosolic DNA sensing to activate the signaling adaptor Stimulator of Interferon Genes (STING), a potent IFNβ inducer (2, 3). The paradigm that DNA sensors incite immunity via STING/IFN-I signaling was also reinforced by reports showing that cyclic dinucleotides (CDNs), which bind and activate STING, incited robust anti-tumor immunity (4). Consequently, STING agonists have emerged as promising adjuvants to treat infections and cancer.

On the other hand, IFN-I also induces specialized DC subsets to express indoleamine 2,3 dioxygenase (IDO), which acquire potent T cell regulatory functions due to increased tryptophan (Trp) metabolism (5). IDO activity is commonly elevated at sites of chronic inflammation associated with AI disease, suggesting that IFN-I signaling may have diametric roles in regulating, as well as promoting autoimmunity. Consistent with this paradigm, STING/IFN-I signaling slowed autoimmune lupus disease progression in susceptible MRL^lpr^ mice and promoted tumor growth (6–8). Moreover, cargo DNA released by DNA nanoparticles (DNPs) and CDNs alleviated experimentally induced autoimmune encephalitis (EAE) and arthritis in mice by stimulating STING/IFN-I signaling to induce IDO1 gene expression (9, 10). Increased IDO activity suppresses immunity by depleting tryptophan (Trp) to activate GCN2 stress responses and generating immune suppressive Trp metabolites, some of which stimulate aryl hydrocarbon receptor (AhR) signaling (11). Therapeutic responses to DNPs and apoptotic cells were dependent on STING/IFN-I signaling to induce IDO in specific DC subsets to suppress effector T cell responses (12). Thus, STING/IFN-I signaling incites diametric immune responses in distinct settings of disease and therapy. Here we show that therapeutic responses to DNPs in the EAE model depend on the cytosolic DNA sensor cGAS and the PD-1/PD-L pathway. Moreover, DNP treatments to activate STING were more effective and durable when combined with drugs that modify Trp metabolism downstream of IDO.

## Materials and Methods

### Mice and Induction of EAE

C57BL/6J (B6) and PD1-deficient (PD1-KO) mice were obtained from Jackson Laboratories, PD-L2-KO, PD-L1/2-KO were kindly provided by Arlene Sharpe (Harvard Medical School, MA, USA) and cGAS-KO mice were kindly provided by Herbert (Skip) Virgin (Washington University, St. Louis, MO). Mice were bred under specific pathogen-free conditions at Augusta University, USA or at Newcastle University, UK. ARRIVE guidelines were followed to minimize the number of animals used.

### EAE Induction and Treatments

To induce EAE, mice (age 8–12 wk) were immunized at two sites on rear flanks with myelin oligodendrocyte glycoprotein peptide MEVGWYRSPFSRVVHLYRNGK (MOG_35−55_; 100 μg, s/c, Bio Basic Canada) emulsified in CFA (Difco Laboratories, Detroit, MI) containing 4 mg/ml *Mycobacterium tuberculosis* H37Ra (Difco Laboratories). Pertussis toxin (PTX, 200 ng; Sigma-Aldrich) was given (i/p) on days 0 and 2 after immunization. Clinical symptoms of EAE were scored on a scale from 0 to 5 as follows: 0, no clinical signs; 0.5, partial limp tail; 1, full limp tail or waddling gait; 1.5, limp tail and waddling gait/lumbar weakness; 2, partial paralysis of one hindlimb; 2.5, paralysis of one hindlimb or partial paralysis of both hindlimbs; 3, paralysis of one hindlimb and partial paralysis of the other hindlimb; 4, paralysis of both hindlimbs; 5, complete paralysis of both hindlimbs and weakness of the upper limb/three paralyzed limbs/moribund state (>20% weight loss and/or complete hind paralysis with hyperventilation and urinary incontinence) or dead. By disease onset groups of mice received intravenous injections of DNPs (prepared by mixing 7 μl of 150 mM linear polyethylenimine, PEI, from Sigma, with 21 μg CpG^free^ LacZ pDNA in 200 μl 5% glucose solution, N/P = 16.7), or cyclic guanylate (2′,5′) monophosphate/adenosine (3′,5′) monophosphate (cGAMP; Invivogen; 100 μg/200 ul saline) or vehicle treatments. Some mice received the following drugs; Kat II inhibitor, PF-04859989 (Sigma, given daily from day 11–22, 40 mg/kg, i/p), AhR inhibitor CH-223191 (Sigma; 5 mg/kg, i/p given 1 h before each cGAMP injection), HAAO inhibitor, 4-chloro-3-hydroxyanthranilic acid (Enamine given daily from day 11–21, 125 mg/kg, i/p).

### IDO Enzyme Assay

IDO activity was measured by high performance liquid chromatography (HPLC) as described (13). Briefly, cells were harvested, lysed (2 × freeze/thaw) and incubated in substrate mixture (100 mM potassium phosphate buffer pH = 6 containing 50 uM methylene blue, 50 mM ascorbate, 0.4 mM L-Tryptophan, 20 ug catalase; all from Sigma) for 0 or 2 h at 37°C. Reaction was stopped and protein precipitated with 1:10 perchloric acid 60% (Sigma); samples were centrifuged, filtered, and the kynurenine levels measured by HPLC. Results are expressed as pmol of kynurenine ([2 h]-[0])/hour/mg of protein.

### FACS Staining

Splenocyte suspensions were stained in PBS with antibodies against the surface markers CD11c (clone N418, eBioscience), CD11b (clone M1/70, Biolegend), or CD19 (clone 1D3, BD horizon), PD-L1 (clone 10F.9G2, BD horizon). After washing with PBS, flow cytometric analyses were performed using a Fortessa X20 cytometer (BD Biosciences), and data generated were analyzed using FACSDiva (BD Biosciences) or FlowJo (Tree Star, Ashland, OR) software. All antibody dilutions were used as per manufacture's instruction.

### Extraction of Kynurenine Pathway Metabolites From Tissue Samples

Mouse brain/spleen tissue samples were suspended in ice-cold 0.32 M sucrose solution (~500 μL solution/100 mg tissue) in sterile 2 mL Eppendorf tubes. Tissue samples were maintained on ice throughout the homogenization protocol. Two homogenization beads were added to each sample and the samples disrupted using a TissueLyser instrument (Qiagen) for 2 min at 25 shakes/second. Homogenate samples were centrifuged at 13,000 rpm for 3 min before the supernatant was transferred to a fresh Eppendorf tube. Samples were centrifuged and transferred again, as above. The total protein concentration in each tissue homogenate sample was determined using a Pierce BCA Protein Assay Kit, according to the manufacturer's protocol. Further adjustments to tissue homogenate dilution/concentration were made using ice-cold 0.32M sucrose solution.

### Analysis of Kynurenine Pathway Metabolites by Liquid Chromatography-Tandem Mass Spectrometry (LC-MS/MS)

Biological sample (100 μL; serum or diluted homogenate) were diluted with 4% phosphoric acid (100 μL). A calibration curve for each metabolite was prepared over ranges (0.05–100 ng) to determine metabolite concentrations. Samples and calibration standards were spiked with internal standard (d5-tryptophan) and loaded onto individual wells of a Waters 96-well array HLB solid phase extraction plate pre-conditioned with methanol (500 μL) and water (500 μL). Samples were washed with water (500 μL) and eluted with 70% methanol (200 μL). Reduced to dryness and resuspended in 30% methanol (70 μL). Using a Shimadzu Nexera UPLC system, 10 μL of each sample well was injected (20 μL) onto an ACE C18-PFP (100 mm × 2.1 mm, 1.7 μm, ACT Technologies, Aberdeen, UK) coupled to an ABSciex QTRAP 6500+ mass analyzer operated in polarity switching electrospray mode. Flow rate was set at 0.5 mL/min at 40 °C. A water:methanol gradient (both containing 0.1% formic acid) was used for separation, with the following conditions: 90:10 water:methanol to 20:80 over 6 min, followed by re-equilibration to 80:20. The total run time was 9 min. Analyst quantitation software (ABI Sciex) was used to acquire and process the data, following European Medicines Agency method validation guidelines for accuracy, precision and limits of detection.

### Statistical Analysis

Statistical significance for EAE clinical scores were evaluated with two-way ANOVA, and normality checks were carried out on the residuals which were approximately normally distributed. The unpaired Student *t*-test was used for the remaining statistical evaluations after normality was verified by the Shapiro-Wilk test. Two-tailed *p* values < 0.05 were considered significant. Data represent mean ± SEM. GraphPad Prism was used to perform all data analyses.

### Study Approval

All procedures were approved by the local Institutional Animal Care and Use Committee in Augusta University and the UK Home Office (Project license P1B4042BB).

## Results

### Therapeutic Responses to DNPs Depend on cGAS

**Figure 1:**
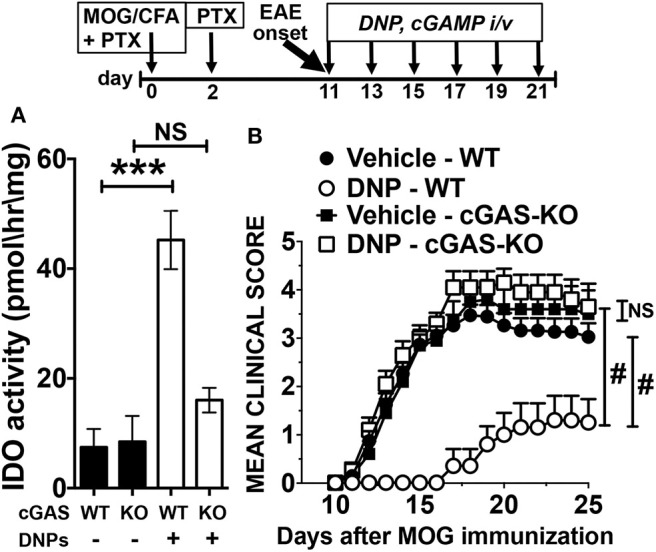
The DNA sensor cGAS mediates therapeutic responses to DNPs that suppress EAE. **(A)** IDO activity in spleens from WT and cGAS-KO (KO) mice treated with DNPs. **(B)** EAE was induced in B6 (WT) and cGAS-KO mice (see section Materials and Methods), and EAE progression was monitored and scored. Experimental groups (*n* = 10) were treated with DNPs (every other day from day 11–21) and control WT mice were treated with vehicle (glucose 5%). Data were analyzed by *t*-test for **(A)** and two-way ANOVA for **(B)**. Experiments were repeated once. NS, not significant; ***p < 0.001, ^#^*p* < 0.0001.

As we reported previously, systemic (i/v) DNP administration elevated IDO activity in many mouse tissues (9). DNPs did not induce IDO activity in spleens from mice lacking the cytosolic DNA sensor cGAS (Figure 1A). Consistent with this finding, DNP treatment from EAE onset (11 days after MOG immunization until day 21) had no significant effect on EAE progression, while cGAS ablation had no effect on EAE progression *per se* (Figure 1B). As expected, DNPs reduced EAE clinical scores in B6 mice significantly until experimental endpoints at day 25. Thus, DNP cargo DNA is sensed by cGAS to induce tolerogenic responses that attenuate EAE progression.

### Therapeutic Responses to STING Agonists Depend on the PD-1/PD-L Pathway

**Figure 2:**
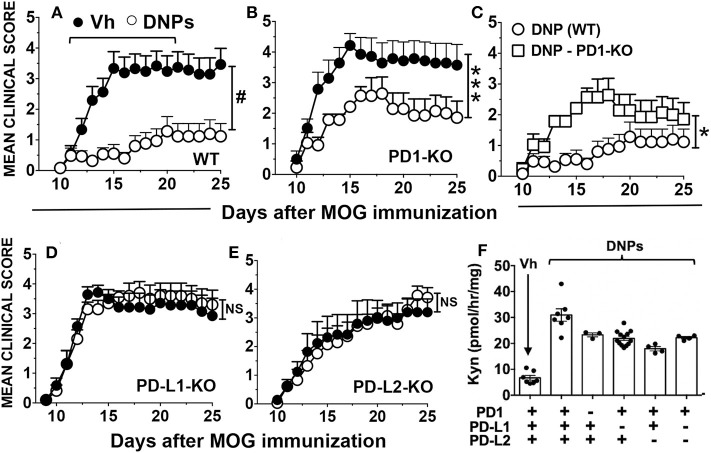
Therapeutic responses to DNPs depend on the PD-1/PD-L pathway. **(A–E)** EAE was induced in B6 (WT), PD-1-KO **(B,C)**, PD-L1-KO **(D)**, and PD-L2-KO **(E)** mice, and disease onset and progression was monitored and scored. At EAE onset (day 11) mice were treated with DNPs every other day until day 21 and control mice were treated with vehicle (Vh; glucose 5%). Data were analyzed by two-way ANOVA (NS, not significant; *p < 0.05; ***p < 0.001, ^#^*p* < 0.0001) and are representative of 2 experiments with *n* = 6–18. **(F)** B6 (WT), PD-1-KO, PD-L1-KO, PD-L2-KO and PD-L1+L2-KO mice were treated with DNPs or Vh (glucose 5%). Twenty-four hours later spleens were harvested and IDO activity in homogenized tissues was measured by assessing Kyn production *ex vivo*. Kyn levels were significantly higher (*p* < 0.0001, *n* = 4–9, Student's *t*-test) in spleens from all mice treated with DNPs regardless of their genotype, relative to Kyn levels in spleens from vehicle-treated WT mice.

The PD-1/PD-L pathway is required for tolerogenic DCs expressing IDO1 to activate Foxp3-lineage regulatory CD4 T cells (Tregs), which then suppress effector T cell responses via a mechanism dependent on PD-1/PD-L signaling (14, 15). To test if the PD-1/PD-L pathway was required for therapeutic responses to DNPs, EAE was induced in mice lacking genes encoding PD-1 or its ligands (PD-L1, PD-L2) and mice were treated with DNPs at the time of EAE onset (Figure 2). Ablating PD-1 or its ligands had no significant impact on the kinetics of EAE induction or clinical severity, relative to WT control mice, though EAE progression was slightly slower in PD-L2-KO mice (Figure 2E). PD-1 ablation did not abolish therapeutic responses to DNPs (Figure 2B) but EAE clinical severity was significantly higher (scores 2–3) in DNP-treated PD-1-KO mice (Figure 2C), relative to DNP-treated WT control mice (scores ~1). Moreover, EAE severity in DNP-treated PD-1-KO mice was only slightly lower than in vehicle-treated WT mice (scores 3–4). In contrast, therapeutic responses to DNPs that slowed EAE progression and reduced clinical severity were abolished in mice lacking PD-L1 (Figure 2D), PD-L2 (Figure 2E) or both PD-1 ligands (Supplemental Figure 1A). Thus, PD-L1 and PD-L2 have non-redundant roles in mediating tolerogenic responses to DNPs that alleviate EAE.

To test if PD-1/PD-L signaling was required to induce IDO1 in splenic DCs we assessed IDO enzyme activity in spleens of naïve mice with defective PD-1/PD-L pathways 24 h after DNP treatment. Following STING activation, induced IDO activity in spleens of WT mice and mice with defective PD-1/PD-L signaling pathways was significantly higher than basal levels in untreated mice, though induced IDO activity was slightly lower in the absence of functional PD-1/PD-L signaling (Figure 2F). Thus, therapeutic responses to DNP treatment were dependent on IDO induction and PD-1/PD-L signaling but IDO1 induction was not dependent on PD-1/PD-L signaling.

**Figure 3:**
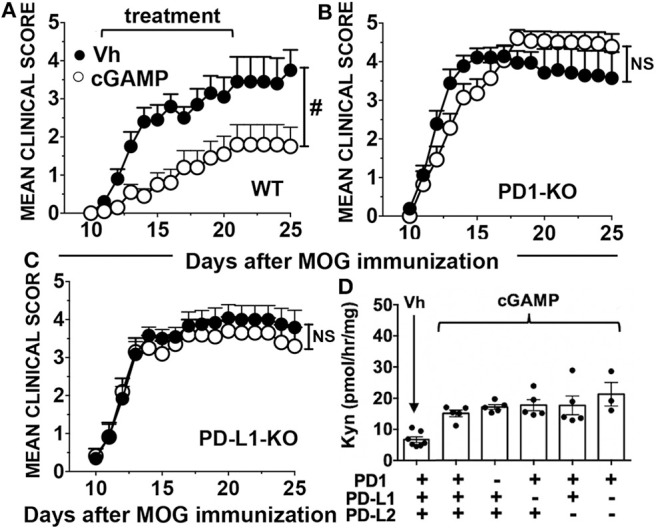
The PD-1/PD-L pathway is required for therapeutic responses to cGAMP. **(A–C)** EAE was induced in B6 (WT), PD-1-KO, and PD-L1-KO mice, and disease onset and progression was monitored and scored. At EAE onset (day 11) mice were treated with cGAMP every other day until day 21 and control mice were treated with vehicle (Vh; saline). Data were analyzed by two-way ANOVA (NS, not significant; #*p* < 0.0001) and are representative of 2 experiments with *n* = 6–18. **(D)** Mice were treated with cGAMP or Vh (saline) and 24 h later spleens were harvested and IDO activity in homogenized tissues was measured by assessing Kyn production *ex vivo*. Data were analyzed using Student's *t*-test and Kyn levels were significantly higher in all mice (*n* = 4–9) treated with cGAMP, B6 (WT, *p* < 0.001), PD-1-KO (*p* < 0.0001), PD-L1-KO (*p* < 0.01), PD-L2-KO (*p* < 0.05) and PD-L1+L2-KO (*p* < 0.05) mice relative to basal Kyn levels in vehicle-treated WT mice.

Cyclic 2′3′-guanyl-adenyl monophosphate (cGAMP) is a potent natural STING agonist made when cGAS is activated by DNA in mammalian cells (16). We tested if synthetic cGAMP treatments incited tolerogenic responses that slow EAE progression. cGAMP treatment from the time of EAE onset (days 11–21) reduced clinical scores significantly until experimental endpoints at day 25 (Figure 3A). As for DNPs, therapeutic responses to cGAMP were abolished in mice lacking PD-1 or PD-L1 genes (Figures 3B,C) or genes encoding both PD-1 ligands (Supplemental Figure 1B). In addition, IDO1 induction in spleen was not dependent on PD-1/PD-L signaling after cGAMP treatment to activate STING (Figure 3D).

### DNPs Enhance PD-L1 Expression by Splenic APCs

**Figure 4:**
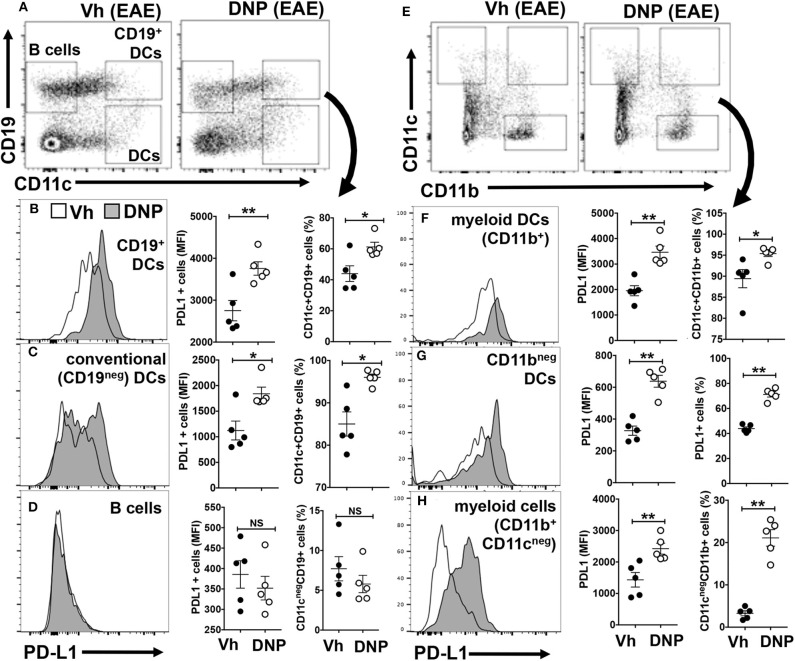
DNPs enhance maturation and expansion of tolerogenic APCs expressing PD-L1. EAE was induced in B6 (WT), and experimental groups were treated with either DNP or its vehicle (Veh; 5% glucose) from day 11–21, every other day. On day 22 splenocytes were harvested and stained for CD11c, CD11b, CD19, and PDL1, and analyzed by flow cytometry. Gating strategy and percentages of the populations **(A,E)** analyzed for PDL1. Percentages of different populations of DCs and monocytes expressing PDL1 and the levels of PDL1 expression are shown **(B–D,F–H)**. Statistical significance was determined by two tailed unpaired Student's *t*-test; NS, not significant; *p < 0.05; **p < 0.01.

APCs expressing PD-L1 suppress effector T cell responses and stabilize the regulatory functions of Foxp3 in Tregs via PD-1 (17, 18). As EAE induction alters APC phenotypes and numbers we examined splenic APCs from mice treated with vehicle (Vh, 5% glucose) or DNPs 22 days after MOG immunization. DNP treatment stimulated splenic DCs (CD11c^+^) and myeloid (CD11b^+^) cell subsets to up-regulate PD-L1 expression, while B cells remained PD-L1^neg^ (Figure 4). PD-L1 expression increased uniformly on a small population of splenic DCs (CD11c^+^) co-expressing the B cell marker CD19 (CD19^+^ DCs), which also expanded following DNP treatment (Figure 4B). As reported previously, CD19^+^ DCs exhibit hybrid features of both B cells and DCs, and amongst splenic DC subsets, CD19^+^ DCs are uniquely competent to express IDO1 (19). PD-L1 expression also increased on a subset (~50%) of conventional (CD19^neg^) DCs, which also expanded after DNP treatment (Figure 4C). In contrast, PD-L1 expression did not increase on B cells, nor did B cells expand after DNP treatment (Figure 4D). Myeloid (CD11b^+^) cells, as well as many CD11b^+^ and CD11b^neg^ DCs, also expressed elevated PD-L1 and expanded following DNP treatment (Figures 4F–H). DNP treatment did not enhance MHCII levels significantly on APCs during EAE (Supplemental Figure 2), though even in naïve mice CD19^+^ DCs expressed the highest levels of MHCII, consistent with the mature APC status of this discrete DC subset (20). Thus, DNP treatment promoted regulatory functions and expansion of DCs but not B cells in mice with EAE.

### Trp Depletion and AhR Signaling Are Dispensable for Therapeutic Responses After STING Activation

IDO activity depletes Trp to induce the integrated stress response (ISR) driven by ribosomal GCN2 kinase, which shuts down most protein synthesis but induces C/EBP homologous protein (CHOP) expression (21). The ISR blocks effector T cell responses, activates Tregs and suppresses pro-inflammatory IL-6 production by plasmacytoid DCs (pDCs) to suppress immunity (22, 23). Previous reports showed that GCN2 is not required for EAE progression but is required for partial EAE remission to manifest after peak disease (24, 25). We tested if GCN2 and CHOP were required for therapeutic responses to manifest after STING agonist treatments. Therapeutic responses to cGAMP were comparable in WT and GCN2-deficient (GCN2-KO) mice (Supplemental Figure 3A), indicating that GCN2 is not essential for therapeutic responses following STING activation to boost IDO activity. Therapeutic responses to DNPs were also comparable in CHOP-KO and WT mice (Supplemental Figure 3B). These outcomes indicate that GCN2 and CHOP are both dispensable for therapeutic responses following STING activation.

Some Trp metabolites such as kynurenine (Kyn), suppress immunity via AhR signaling (26, 27). Previous reports showed that AhR gene ablation had minor or no significant effects on EAE susceptibility, while treatments to block AhR signaling suppressed EAE in WT mice (28–32). Prompted by this prior work, we tested if AhR signaling mediated therapeutic responses to STING agonists. Mice were given the AhR antagonist drug CH223191 (CH) at optimal doses (5 mg/kg) shown to block AhR signaling effectively in mice (30, 33–35). CH treatments were administered daily from EAE onset (day 11). Therapeutic responses to cGAMP were comparable in the presence or absence of CH co-treatments (Supplemental Figure 3C), suggesting that AhR signaling is dispensable for therapeutic responses induced by cGAMP.

### Manipulating Trp Catabolism Potentiates Therapeutic Responses to DNPs

**Figure 5:**
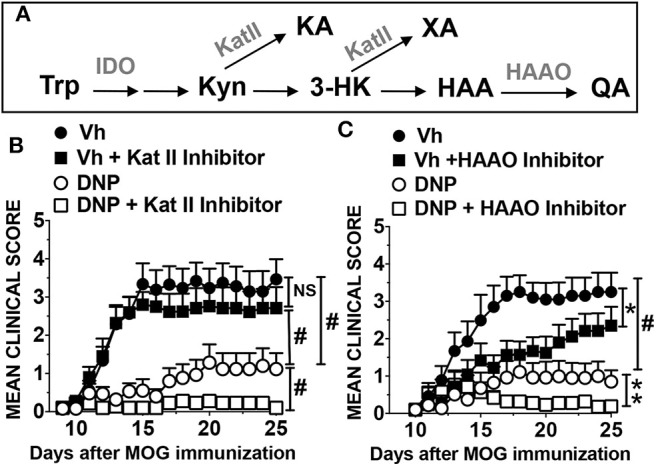
Manipulating the IDO pathway reinforces therapeutic responses to DNP treatments. **(A)** The kynurenine pathway. **(B,C)** B6 (WT) mice were treated with DNPs or Vh (glucose 5%), as described previously. Other groups received DNPs and drugs that inhibit KatII (**B**; PF-04859989 daily from day 11–22, 40 mg/kg, i/p), or HAAO (**C**; 4-chloro-3-hydroxyanthranilic acid daily from day 11–21, 125 mg/kg, i/p). Mice were monitored and EAE clinical scores were recorded. Data were analyzed by two-way ANOVA. Experiments were repeated once (*n* = 8–10). NS, not significant; *p < 0.05; **p < 0.01; #*p* < 0.0001.

In addition to immunosuppressive Trp metabolites, cells expressing IDO1 may produce neuroactive Trp metabolites that exacerbate comorbidities such as pain, depression, and fatigue in patients with MS and other neuroinflammatory syndromes (36–38). Quinolinic acid (QA) is a N-methyl-D-aspartate (NMDA) receptor agonist and sustained production of neuro-excitatory QA in the central nervous system (CNS) causes neuronal injury. Another Trp catabolite, kynurenic acid (KA), antagonizes NMDA receptor signaling and the KA:QA balance is a pivotal factor modulating neurologic comorbidities (39). Since increased IDO activity may have neurologic consequences such as increased nociception (40), we examined how drugs that block production of neuroactive QA or KA impact therapeutic responses to DNPs in the EAE model. As depicted in Figure 5A, kynurenine aminotransferase-II (KatII) converts Kyn into KA and 3-hydroxyanthranilic acid (HAA) dioxygenase (HAAO) converts HAA into QA. The KatII inhibitor PF-04859989 (PF) had no significant impact on EAE progression or clinical severity when applied at EAE onset (Figure 5B). Remarkably, when combined PF and DNP treatments were administered at EAE onset most mice showed no symptoms of EAE (mean score <0.2) during or after treatments (Figure 5B). Therapeutic responses to combined PF and DNPs were significantly better than responses to DNPs (mean score 1). Thus, PF and DNP co-treatments suppressed EAE progression in the majority of mice, with mild symptoms persisting in only a few mice in this group. Similar outcomes were observed in mice co-treated with an HAAO inhibitor, 4-chloro-3-hydroxyanthranilic acid (4Cl-HAA), though 4Cl-HAA monotherapy slowed EAE progression significantly but did not prevent EAE progression (Figure 5C). Thus, KatII inhibition synergized with DNP treatments while HAAO inhibition supplemented therapeutic responses to DNPs to suppress EAE progression. Thus, STING activation to boost IDO activity in the presence of KatII or HAAO inhibitors are effective strategies to suppress EAE progression.

Targeted metabolomics (LC-MS/MS) analyses (41, 42) of brain tissues taken at peak EAE severity (day 14, 3 h after final treatments) revealed that DNP treatments boosted brain Kyn, relative to levels in mice with EAE, and that increased brain Kyn correlated with reduced EAE severity (Supplemental Figure 4A). Elevated Kyn was also detected in spleen and serum of DNP-treated mice with EAE (data not shown) confirming that DNPs boost IDO activity systemically. However, DNP treatments reduced brain IDO expression in neurons of MOG-immunized mice (10), suggesting that increased brain Kyn in DNP-treated mice may be generated in non-CNS tissues and crosses the blood-brain barrier. DNP treatments reduced brain 3HAA levels significantly (Supplemental Figure 4B) and boosted brain 3HK levels, albeit not significantly (Supplemental Figure 4C), while brain KA levels were unaffected by EAE induction or DNP treatments (Supplemental Figure 4D). Co-treatment with KatII inhibitor (PF) blocked DNP-induced increase in brain Kyn, while brain Kyn levels were still elevated in mice co-treated with HAAO inhibitor (4Cl-HAA) and DNPs (Supplemental Figure 4A). Further analyses of metabolomics data provided no fresh insights into underlying metabolic processes that explain substantial differences in EAE severity in distinct treatment groups.

## Discussion

Previously, we showed that DNPs and a bacterial CDN, cyclic diguanyl monophosphate (cdiGMP), activated STING to enhance IDO-dependent regulatory functions of splenic DCs and suppress antigen induced arthritis (AIA) and EAE in mice (9, 10, 12). In this study, we further dissect critical pathway components that promote therapeutic responses to STING agonists in the EAE model and show that drugs targeting KatII or HAAO reinforce therapeutic responses to DNPs to suppress EAE progression.

The finding that the cytosolic DNA sensor cGAS promotes therapeutic responses and is required to induce IDO after DNP treatment reveals that cGAS senses DNP cargo DNA to activate STING/IFN-I signaling in DCs competent to express IDO and suppress autoimmunity. STING also promoted tolerogenic responses that attenuated autoimmune lupus progression in susceptible MRL^lpr^ mice, suggesting that cytosolic DNA sensors promote tolerogenic responses that slow lupus progression in this model (7), though specific requirements for cGAS or IDO to promote tolerogenic responses were not addressed in this study. STING agonists also induced tolerogenic responses that promoted Lewis Lung Carcinoma (LLC) growth in mice by stimulating IDO activity in inflamed lymph nodes draining sites of tumor growth, though cGAS was not essential to induce IDO1 in the LLC model (8). In contrast, STING agonists incited potent anti-tumor responses in mice bearing more immunogenic B16 melanomas (4). Opposing outcomes in distinct tumor models emphasize that STING signaling may stimulate or suppress immunity. Elevated IDO activity is a common immune checkpoint in cancer (43), reinforcing the paradigm that boosting IDO activity protects inflamed tissues from immune-mediated destruction. On the other hand, cytosolic DNA sensing by cGAS to activate STING incited anti-viral immunity, spontaneous autoimmunity in mice with defective DNA catabolizing enzymes and anti-tumor immunity (44–48). How DNA from cellular, microbial or artificial sources incites diametric tolerogenic or immunogenic responses is unclear. Differential DNA uptake and sensing by distinct cell types in different tissues may be key factors driving diametric immune responses to DNA in different inflammatory settings. Sustained tissue re-modeling and local inflammation enhance cell death and dying cells promote potent tolerogenic responses, in part because DNA from dying cells is sensed to induce IDO1 and suppress immunity and autoimmunity (12, 49, 50). Intra-tumoral injection of synthetic CDNs was essential to incite robust anti-tumor responses to immunogenic B16 melanomas (4). In contrast, systemic administration of STING agonists was necessary to induce tolerogenic responses (9, 10, 12). These findings suggest that circulating STING agonists and dying cells are sensed to induce tolerogenic processes that protect tissues from immune-mediated attack, while DNA sensing to activate STING in inflamed tissues may incite immunogenic or tolerogenic responses that protect against infections or promote tumor growth, respectively. Previously, we identified splenic myeloid (CD11b^+^) DCs as cells uniquely competent to incite IDO1-dependent tolerogenic responses by ingesting and sensing DNP cargo DNA or CDNs to activate STING/IFN-I signaling and induce specialized splenic DCs to express IDO1 (12). Thus, splenic myeloid DCs may be pivotal cells that sense ingested DNA and cGAMP to generate robust tolerogenic responses that prevent EAE progression.

PD-1/PD-L signaling drives potent tolerogenic responses that suppress effector T cells and activate Tregs in multiple settings (51). Therapeutic responses to DNPs in the EAE model were partially dependent on PD-1 and fully dependent on PD-L1 and PD-L2, respectively, while therapeutic responses to cGAMP were abolished in mice lacking PD-1 or PD-L1. It is unclear how PD-1 independent therapeutic responses to DNPs manifest but DNPs contain the cationic polymer polyethylenimine that stimulates innate immune signaling via TLR5 (52), while cGAMP targets STING only. STING agonist treatments enhanced the numbers of splenic DCs (CD11c^+^) and myeloid (CD11b^+^) cells expressing PD-L1 and PD-L1 levels expressed by these cells, suggesting that DCs and myeloid antigen presenting cells (APCs) suppress T cell responses driving EAE. Potential roles for autoreactive B cells in MS immunopathology have been proposed because B cell depleting anti-CD19 mAbs alleviated MS in some patients (53). Discrete B cell subsets also promote or regulate EAE in mice (54, 55). In mice, a discrete subset of marginal zone DCs co-expressing the B cell marker CD19 (CD19^+^ DCs) are uniquely competent to express IDO1 and become potent regulatory APCs (19). CD19^+^ DCs expressing IDO1 suppress T cell responses and activate Tregs to block their conversion into T_H_17 T cells. Phenotypic overlaps between CD19^+^ DCs and B cells may explain why some ‘B cell’ subsets regulate EAE. CD19^+^ DCs expressing IDO1 also expanded in spleens of aged lupus-prone Nba2 mice and during chronic retroviral infections in mice (40, 56). Thus, regulatory B cell subsets, CD19^+^ DCs expressing IDO1 and other regulatory APCs may contribute to therapeutic responses to STING agonists that suppress EAE.

Defects in PD-1/PD-L signaling did not compromise IDO1 induction following STING activation, indicating that the PD-1/PD-L and IDO1 pathways act independently to promote tolerogenic responses in response to STING activation. In contrast, PD-1 signaling was essential to induce IDO1 and tolerogenic responses following TLR9 ligation (15). Like STING agonists, TLR9 ligands stimulate IFN-I production by DCs and IFN-I induces IDO (17, 19). Ablating CD19 or the PD-1/PD-L pathway abolished IDO induction in CD19^+^ DCs and tolerogenic responses following TLR9 ligation and instead elicited immunogenic responses. Thus, CD19^+^ DCs are pivotal cells controlling responses to TLR9 ligands and PD-1/PD-L interactions between Tregs (or T cells) and CD19^+^ DCs are necessary to induce CD19^+^ DCs to express IDO1 and acquire tolerogenic phenotypes in this setting. As IDO induction after STING activation was not PD-1/PD-L dependent, signals from Tregs or T cells expressing PD-1 to induce CD19^+^ DCs to express IDO1 are not required for tolerogenic responses to STING agonists. It is unclear why PD-1/PD-L interactions were not required to induce IDO1 in CD19^+^ DCs following STING activation. However, downstream interactions between regulatory APCs or other cells types expressing PD-1 ligands and T cells or Tregs expressing PD-1 are essential to promote tolerogenic responses that suppress EAE.

IDO activity inhibits T cell responses via two metabolic pathways, Trp depletion to activate the GCN2/CHOP-dependent ISR and Trp catabolite production (11, 57). HAA suppresses T cell responses by inhibiting PDK1-mediated NF-kB activation to promote T cell apoptosis (58), while Kyn and KA are natural AhR ligands and AhR signaling regulates T cell responses (26, 27, 59). Therapeutic responses to DNPs were unaffected by GCN2 ablation while therapeutic responses to cGAMP were unaffected by AhR signaling blockade, indicating that neither pathway is essential for therapeutic responses to STING agonists. Also, CHOP ablation had no major impact on therapeutic responses to DNPs. Though CHOP is a GCN2 target, other stress pathways can activate CHOP and these alternative pathways may mediate therapeutic effects. The ISR and AhR signaling may be dispensable for therapeutic responses to STING agonists because each pathway compensates for loss of the other making each pathway functionally redundant for therapeutic responses. Alternatively, other metabolic effects of elevated IDO activity such as altered redox potentials (60) may influence EAE pathogenesis and therapeutic responses to STING agonists.

Two well-characterized and well-tolerated drugs that inhibit KatII (61–63) or HAAO (64–66) were used to assess metabolic requirements that promote therapeutic responses to DNPs downstream of IDO. Combining either drug with DNPs prevented EAE progression, even after treatments ended. Stable disease control is difficult to achieve in the EAE model, as depots of autoantigen and adjuvant (MOG/CFA) with potent encephalitogenic properties persist at sites of immunization. As KatII converts Kyn into KA and HAAO converts HAA into QA (Figure 5A), our findings suggest that KatII and HAAO activity may attenuate therapeutic responses to DNPs by consuming Trp metabolites that mediate immune suppression such as Kyn, 3-HK and HAA. Previous studies showed that HAA (or a synthetic HAA derivative) attenuated EAE, though therapeutic responses were weak (67, 68). Thus, boosting HAA production and blocking HAA consumption in tissues may be more effective than administering exogenous HAA. Reduced KA or QA production may also contribute to robust therapeutic responses after DNP treatment. HAAO inhibition may attenuate neurologic comorbidities by reducing production of neuroexcitatory QA, though blocking KA production may potentiate neurologic comorbidities since KA antagonizes QA-mediated neuronal stimulation via NMDA. To further evaluate metabolic changes in the Kyn pathway we conducted metabolomics analyses of tissues (brain, spleen) and serum samples. Outcomes confirmed that DNPs boost IDO activity systemically and suggest that Kyn generated in non-CNS tissues following DNP treatment may cross the blood-brain barrier in DNP-treated mice. However, no additional insights were forthcoming, highlighting the technical difficulty of identifying critical metabolic changes in small cohorts of physiologic cells that drive therapeutic responses using metabolomics analyses. These points notwithstanding, our findings show that boosting kinetic flux through the Kyn pathway and manipulating the balance of alternative Trp metabolism pathways downstream of IDO have profound effects on immunologic responses to STING agonists that promote durable therapeutic responses in the EAE model.

**Figure 6:**
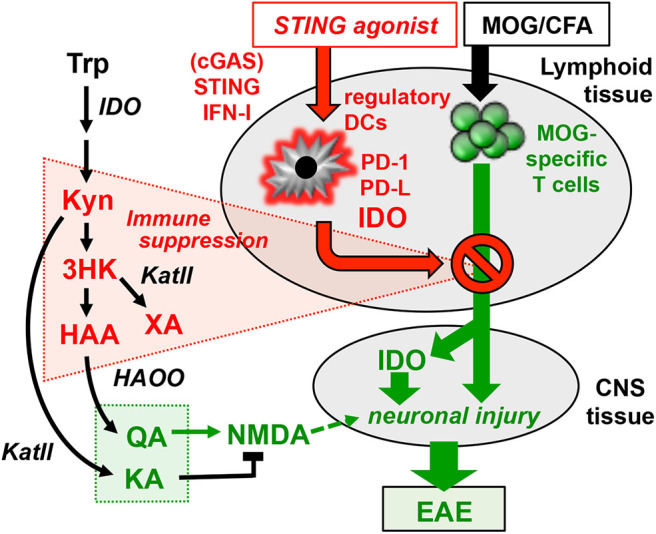
Graphical hypothesis. MOG immunization generates MOG-specific effector T cells that migrate to the CNS where they cause neuronal injury and induce neurons to express IDO1 and produce neurotoxic Trp catabolites such as QA (green highlights). STING agonists induce DCs in lymphoid tissues to express IDO1 and produce Trp catabolites that suppress generation of MOG-specific T cells (red highlights). Manipulating Trp catabolism to enhance production of immune suppressive catabolites and block production of neurotoxic catabolites may be an effective strategy to boost tolerogenic responses that promote stable EAE remission and alleviate neurologic comorbidities.

Abnormal levels of KA and QA were detected in serum of MS patients and abnormalities in Kyn pathway metabolism may be associated with clinical MS subtypes, and may serve as potential biomarkers of MS stage and progression (38, 69, 70). As we depict in the graphic hypothesis (Figure 6) summarizing findings reported here, and in a previous study using the same model (10), the Kyn pathway has a complex role in immunologic and neurologic processes driving autoimmunity and comorbidities during EAE disease progression and responses to STING agonist treatments to alleviate EAE. Sustained IDO expression in CNS tissues contributes to EAE pathogenesis because Trp metabolites such as QA may cause neuro-inflammatory injury leading to psychotic disorders (71). Previously, we reported that EAE induction *per se* stimulates IDO1 expression by CNS neurons, which may promote neuronal injury (10). Paradoxically, DNP treatments abolished IDO expression in CNS neurons, possibly because increased IDO activity in lymphoid tissues blocked expansion and activation of MOG-specific T cells that would otherwise infiltrate CNS tissues and induce local IDO expression, for example by secreting IFNγ. Several Trp metabolites, including Kyn, KA, 3-HK and HAA, have been implicated in immune regulation as they diminish TCR signaling and enhance AhR signaling in T cells and other immune cells (27, 72). The Trp catabolite xanthurenic acid (XA) may also suppress T cell responses after DNP treatment, as XA inhibits synthesis of the enzyme cofactor tetrahydrobiopterin (BH4) by activated T cells and BH4 is essential for T cell clonal expansion (73). Another mechanism that may promote therapeutic responses to STING agonists is that HAA induces CNS astrocytes to express HO-1, which generates compounds with anti-inflammatory and cytoprotective properties (74). Further studies to identify specific cell types that produce and consume particular Trp metabolites, determine the tissue locations of such cells and define targets of Trp metabolites will be key to understanding how increased metabolic flux through the Kyn pathway contributes to immunologic and neurologic processes during disease progression and comorbidities, as well as responses to therapy. In summary, our findings reveal that boosting IDO activity and manipulating the IDO pathway is an effective therapeutic strategy to treat neuro-inflammatory autoimmune syndromes.

## Data Availability Statement

All datasets generated for this study are included in the article/Supplementary Material.

## Ethics Statement

The animal study was reviewed and approved by IWERB Newcastle University.

## Author Contributions

Research studies were designed by HL, EM, LH, and AM. Experiments were performed and data acquired by HL, EM, LH, CM, RO, XZ, KM, NH, and DM. Data were analyzed by HL, EM, and LH. HL, LH, and AM wrote the manuscript. All authors contributed to the article and approved the submitted version.

## Conflict of Interest

AM holds shares in NewLink Genetics Inc. and receives income from patents licensed to this company. The remaining authors declare that the research was conducted in the absence of any commercial or financial relationships that could be construed as a potential conflict of interest.

## References

[1] Banchereau J Pascual V. Type I interferon in systemic lupus erythematosus and other autoimmune diseases. Immunity. (2006) 25:383–92. 10.1016/j.immuni.2006.08.010 16979570

[2] Ahn J Gutman D Saijo S Barber GN. STING manifests self DNA-dependent inflammatory disease. Proc Natl Acad Sci USA. (2012) 109:19386–91. 10.1073/pnas.1215006109 23132945 PMC3511090

[3] Gall A Treuting P Elkon KB Loo YM Gale M Jr Barber GN. Autoimmunity initiates in nonhematopoietic cells and progresses via lymphocytes in an interferon-dependent autoimmune disease. Immunity. (2012) 36:120–31. 10.1016/j.immuni.2011.11.018 22284419 PMC3269499

[4] Corrales L Glickman LH McWhirter SM Kanne DB Sivick KE Katibah GE. Direct Activation of STING in the tumor microenvironment leads to potent and systemic tumor regression and immunity. Cell Rep. (2015) 11:1018–30. 10.1016/j.celrep.2015.04.031 25959818 PMC4440852

[5] Huang L Baban B Johnson BA Mellor AL. Dendritic cells, indoleamine 2,3 dioxygenase and acquired immune privilege. Int Rev Immunol. (2010) 29:133–55. 10.3109/08830180903349669 20367139 PMC3671384

[6] Ahn J Xia T Konno H Konno K Ruiz P Barber GN. Inflammation-driven carcinogenesis is mediated through STING. Nat Commun. (2014) 5:5166. 10.1038/ncomms6166 25300616 PMC4998973

[7] Sharma S Campbell AM Chan J Schattgen SA Orlowski GM Nayar R. Suppression of systemic autoimmunity by the innate immune adaptor STING. Proc Natl Acad Sci USA. (2015) 112:E710–7. 10.1073/pnas.1420217112 25646421 PMC4343138

[8] Lemos H Mohamed E Huang L Ou R Pacholczyk G Arbab AS. STING promotes the growth of tumors characterized by low antigenicity via IDO activation. Cancer Res. (2016) 76:2076–81. 10.1158/0008-5472.CAN-15-1456 26964621 PMC4873329

[9] Huang L Lemos HP Li L Li M Chandler PR Baban B. Engineering DNA nanoparticles as immunomodulatory reagents that activate regulatory T cells. J Immunol. (2012) 188:4913–20. 10.4049/jimmunol.1103668 22516958 PMC3349160

[10] Lemos H Huang L Chandler PR Mohamed E Souza GS Li L. Activation of the STING adaptor attenuates experimental autoimmune encephalitis. J Immunol. (2014) 192:5571–8. 10.4049/jimmunol.1303258 24799564 PMC4086255

[11] McGaha TL Huang L Lemos H Metz R Mautino M Prendergast GC. Amino acid catabolism: a pivotal regulator of innate and adaptive immunity. Immunol Rev. (2012) 249:135–57. 10.1111/j.1600-065X.2012.01149.x 22889220 PMC4384693

[12] Huang L Li L Lemos H Chandler PR Pacholczyk G Baban B. Cutting edge: DNA sensing via the STING adaptor in myeloid dendritic cells induces potent tolerogenic responses. J Immunol. (2013) 191:3509–13. 10.4049/jimmunol.1301419 23986532 PMC3788571

[13] Hoshi M Saito K Hara A Taguchi A Ohtaki H Tanaka R. The absence of IDO upregulates type I IFN production, resulting in suppression of viral replication in the retrovirus-infected mouse. J Immunol. (2010) 185:3305–12. 10.4049/jimmunol.0901150 20693424

[14] Sharma MD Baban B Chandler P Hou DY Singh N Yagita H. Plasmacytoid dendritic cells from mouse tumor-draining lymph nodes directly activate mature Tregs via IDO. J Clin Invest. (2007) 117:2570–82. 10.1172/JCI31911 17710230 PMC1940240

[15] Baban B Chandler PR Johnson BA III Huang L Li M Sharpe ML. Physiologic control of IDO competence in splenic dendritic cells. J Immunol. (2011) 187:2329–35. 10.4049/jimmunol.1100276 21813777 PMC3556270

[16] Cai X Chiu YH Chen ZJ. The cGAS-cGAMP-STING pathway of cytosolic DNA sensing and signaling. Mol Cell. (2014) 54:289–96. 10.1016/j.molcel.2014.03.040 24766893

[17] Baban B Chandler PR Sharma MD Pihkala J Koni PA Munn DH. IDO activates regulatory T cells and blocks their conversion into Th17-like T cells. J Immunol. (2009) 183:2475–83. 10.4049/jimmunol.0900986 19635913 PMC3677163

[18] Sharma MD Shinde R McGaha TL Huang L Holmgaard RB Wolchok JD. The PTEN pathway in Tregs is a critical driver of the suppressive tumor microenvironment. Sci Adv. (2015) 1:e1500845. 10.1126/sciadv.1500845 26601142 PMC4640592

[19] Johnson BA III Kahler DJ Baban B Chandler PR Kang B Shimoda M. B-lymphoid cells with attributes of dendritic cells regulate T cells via indoleamine 2,3-dioxygenase. Proc Natl Acad Sci USA. (2010) 107:10644–8. 10.1073/pnas.0914347107 20498068 PMC2890795

[20] Baban B Hansen AM Chandler PR Manlapat A Bingaman A Kahler DJ. A minor population of splenic dendritic cells expressing CD19 mediates IDO-dependent T cell suppression via type I IFN signaling following B7 ligation. Int Immunol. (2005) 17:909–19. 10.1093/intimm/dxh271 15967784

[21] Ron D. Translational control in the endoplasmic reticulum stress response. J Clin Invest. (2002) 110:1383–8. 10.1172/JCI16784 12438433 PMC151821

[22] Munn DH Sharma MD Baban B Harding HP Zhang Y Ron D. GCN2 kinase in T cells mediates proliferative arrest and anergy induction in response to indoleamine 2,3-dioxygenase. Immunity. (2005) 22:1–10. 10.1016/j.immuni.2005.03.013 15894280

[23] Sharma MD Hou D-Y Liu Y Koni PA Metz R Chandler PR. Indoleamine 2,3-dioxygenase controls conversion of Foxp3+ Tregs to TH17-like cells in tumor-draining lymph nodes. Blood. (2009) 113:6102–11. 10.1182/blood-2008-12-195354 19366986 PMC2699232

[24] Orsini H Araujo LP Maricato JT Guereschi MG Mariano M Castilho BA. GCN2 kinase plays an important role triggering the remission phase of experimental autoimmune encephalomyelitis (EAE) in mice. Brain Behav Immun. (2014) 37:177–86. 10.1016/j.bbi.2013.12.012 24362236

[25] Keil M Sonner JK Lanz TV Oezen I Bunse T Bittner S. General control non-derepressible 2 (GCN2) in T cells controls disease progression of autoimmune neuroinflammation. J Neuroimmunol. (2016) 297:117–26. 10.1016/j.jneuroim.2016.05.014 27397084

[26] Quintana FJ Basso AS Iglesias AH Korn T Farez MF Bettelli E. Control of T(reg) and T(H)17 cell differentiation by the aryl hydrocarbon receptor. Nature. (2008) 453:65–71. 10.1038/nature06880 18362915

[27] Mezrich JD Fechner JH Zhang X Johnson BP Burlingham WJ Bradfield CA. An interaction between kynurenine and the aryl hydrocarbon receptor can generate regulatory T cells. J Immunol. (2010) 185:3190–8. 10.4049/jimmunol.0903670 20720200 PMC2952546

[28] Duarte JH Di Meglio P Hirota K Ahlfors H Stockinger B. Differential influences of the aryl hydrocarbon receptor on Th17 mediated responses *in vitro* and *in vivo*. PLoS ONE. (2013) 8:e79819. 10.1371/journal.pone.0079819 24244565 PMC3828240

[29] Hanieh H Alzahrani A. MicroRNA-132 suppresses autoimmune encephalomyelitis by inducing cholinergic anti-inflammation: a new Ahr-based exploration. Eur J Immunol. (2013) 43:2771–82. 10.1002/eji.201343486 23780851

[30] Rouse M Singh NP Nagarkatti PS Nagarkatti M. Indoles mitigate the development of experimental autoimmune encephalomyelitis by induction of reciprocal differentiation of regulatory T cells and Th17 cells. Br J Pharmacol. (2013) 169:1305–21. 10.1111/bph.12205 23586923 PMC3831710

[31] Berg J Mahmoudjanlou Y Duscha A Massa MG Thone J Esser C. The immunomodulatory effect of laquinimod in CNS autoimmunity is mediated by the aryl hydrocarbon receptor. J Neuroimmunol. (2016) 298:9–15. 10.1016/j.jneuroim.2016.06.003 27609269

[32] Kaye J Piryatinsky V Birnberg T Hingaly T Raymond E Kashi R. Laquinimod arrests experimental autoimmune encephalomyelitis by activating the aryl hydrocarbon receptor. Proc Natl Acad Sci USA. (2016) 113:E6145–52. 10.1073/pnas.1607843113 27671624 PMC5068259

[33] Zhao B Degroot DE Hayashi A He G Denison MS. CH223191 is a ligand-selective antagonist of the Ah (Dioxin) receptor. Toxicol Sci. (2010) 117:393–403. 10.1093/toxsci/kfq217 20634293 PMC2940411

[34] Monteleone I Zorzi F Marafini I Di Fusco D Dinallo V Caruso R. Aryl hydrocarbon receptor-driven signals inhibit collagen synthesis in the gut. Eur J Immunol. (2016) 46:1047–57. 10.1002/eji.201445228 26786786

[35] Shinde R Hezaveh K Halaby MJ Kloetgen A Chakravarthy A da Silva Medina T. Apoptotic cell-induced AhR activity is required for immunological tolerance and suppression of systemic lupus erythematosus in mice and humans. Nat Immunol. (2018) 19:571–82. 10.1038/s41590-018-0107-1 29760532 PMC5976527

[36] Lim CK Brew BJ Sundaram G Guillemin GJ. Understanding the roles of the kynurenine pathway in multiple sclerosis progression. Int J Tryptophan Res. (2010) 3:157–67. 10.4137/ijtr.s4294 22084596 PMC3195238

[37] Lovelace MD Varney B Sundaram G Lennon MJ Lim CK Jacobs K. Recent evidence for an expanded role of the kynurenine pathway of tryptophan metabolism in neurological diseases. Neuropharmacology. (2017) 112(Pt B):373–88. 10.1016/j.neuropharm.2016.03.024 26995730

[38] Bansi J Koliamitra C Bloch W Joisten N Schenk A Watson M. Persons with secondary progressive and relapsing remitting multiple sclerosis reveal different responses of tryptophan metabolism to acute endurance exercise and training. J Neuroimmunol. (2018) 314:101–5. 10.1016/j.jneuroim.2017.12.001 29224960

[39] Anderson G Maes M. TRYCAT pathways link peripheral inflammation, nicotine, somatization and depression in the etiology and course of Parkinson's disease. CNS Neurol Disord Drug Targets. (2014) 13:137–49. 10.2174/18715273113129990082 23844687

[40] Huang L Ou R Rabelo de Souza G Cunha TM Lemos H Mohamed E. Virus infections incite pain hypersensitivity by inducing indoleamine 2,3 dioxygenase. PLoS Pathog. (2016) 12:e1005615. 10.1371/journal.ppat.1005615 27168185 PMC4863962

[41] Skouras C Zheng X Binnie M Homer NZ Murray TB Robertson D. Increased levels of 3-hydroxykynurenine parallel disease severity in human acute pancreatitis. Sci Rep. (2016) 6:33951. 10.1038/srep33951 27669975 PMC5037401

[42] Walker AL Ancellin N Beaufils B Bergeal M Binnie M Bouillot A. Development of a series of kynurenine 3-monooxygenase inhibitors leading to a clinical candidate for the treatment of acute pancreatitis. J Med Chem. (2017) 60:3383–404. 10.1021/acs.jmedchem.7b00055 28398044

[43] Munn DH Mellor AL. IDO in the tumor microenvironment: inflammation, counter-regulation, and tolerance. Trends Immunol. (2016) 37:193–207. 10.1016/j.it.2016.01.002 26839260 PMC4916957

[44] Gao D Wu J Wu YT Du F Aroh C Yan N. Cyclic GMP-AMP synthase is an innate immune sensor of HIV and other retroviruses. Science. (2013) 341:903–6. 10.1126/science.1240933 23929945 PMC3860819

[45] Ablasser A Hemmerling I Schmid-Burgk JL Behrendt R Roers A Hornung V. TREX1 deficiency triggers cell-autonomous immunity in a cGAS-dependent manner. J Immunol. (2014) 192:5993–7. 10.4049/jimmunol.1400737 24813208

[46] Schoggins JW Macduff DA Imanaka N Gainey MD Shrestha B Eitson JL. Pan-viral specificity of IFN-induced genes reveals new roles for cGAS in innate immunity. Nature. (2014) 505:691–5. 10.1038/nature12862 24284630 PMC4077721

[47] Gao D Li T Li XD Chen X Li QZ Wight-Carter M. Activation of cyclic GMP-AMP synthase by self-DNA causes autoimmune diseases. Proc Natl Acad Sci USA. (2015) 112:E5699–705. 10.1073/pnas.1516465112 26371324 PMC4620884

[48] Li T Cheng H Yuan H Xu Q Shu C Zhang Y. Antitumor activity of cGAMP via stimulation of cGAS-cGAMP-STING-IRF3 mediated innate immune response. Sci Rep. (2016) 6:19049. 10.1038/srep19049 26754564 PMC4709567

[49] Ravishankar B Liu H Shinde R Chandler P Baban B Tanaka M. Tolerance to apoptotic cells is regulated by indoleamine 2,3-dioxygenase. Proc Natl Acad Sci USA. (2012) 109:3909–14. 10.1073/pnas.1117736109 22355111 PMC3309765

[50] Ravishankar B Shinde R Liu H Chaudhary K Bradley J Lemos HP. Marginal zone CD169+ macrophages coordinate apoptotic cell-driven cellular recruitment and tolerance. Proc Natl Acad Sci USA. (2014) 111:4215–20. 10.1073/pnas.1320924111 24591636 PMC3964059

[51] Francisco LM Sage PT Sharpe AH. The PD-1 pathway in tolerance and autoimmunity. Immunol Rev. (2010) 236:219–42. 10.1111/j.1600-065X.2010.00923.x 20636820 PMC2919275

[52] Cubillos-Ruiz JR Engle X Scarlett UK Martinez D Barber A Elgueta R. Polyethylenimine-based siRNA nanocomplexes reprogram tumor-associated dendritic cells via TLR5 to elicit therapeutic antitumor immunity. J Clin Invest. (2009) 119:2231–44. 10.1172/JCI37716 19620771 PMC2719935

[53] Chen D Gallagher S Monson NL Herbst R Wang Y. Inebilizumab, a B cell-depleting anti-CD19 antibody for the treatment of autoimmune neurological diseases: insights from preclinical studies. J Clin Med. (2016) 5:107. 10.3390/jcm5120107 27886126 PMC5184780

[54] Matsushita T Fujimoto M Hasegawa M Komura K Takehara K Tedder TF. Inhibitory role of CD19 in the progression of experimental autoimmune encephalomyelitis by regulating cytokine response. Am J Pathol. (2006) 168:812–21. 16507897 10.2353/ajpath.2006.050923 PMC1606513

[55] Matsushita T Yanaba K Bouaziz JD Fujimoto M Tedder TF. Regulatory B cells inhibit EAE initiation in mice while other B cells promote disease progression. J Clin Invest. (2008) 118:3420–30. 10.1172/JCI36030 18802481 PMC2542851

[56] Jorgensen TN Alfaro J Enriquez HL Jiang C Loo WM Atencio S. Development of murine lupus involves the combined genetic contribution of the SLAM and FcgammaR intervals within the Nba2 autoimmune susceptibility locus. J Immunol. (2010) 184:775–86. 10.4049/jimmunol.0901322 20018631 PMC2841050

[57] Munn DH Mellor AL. Indoleamine 2,3 dioxygenase and metabolic control of immune responses. Trends Immunol. (2012) 34:137–43. 10.1016/j.it.2012.10.001 23103127 PMC3594632

[58] Hayashi T Mo JH Gong X Rossetto C Jang A Beck L. 3-Hydroxyanthranilic acid inhibits PDK1 activation and suppresses experimental asthma by inducing T cell apoptosis. Proc Natl Acad Sci USA. (2007) 104:18619–24. 10.1073/pnas.0709261104 18003900 PMC2141826

[59] DiNatale BC Murray IA Schroeder JC Flaveny CA Lahoti TS Laurenzana EM. Kynurenic acid is a potent endogenous aryl hydrocarbon receptor ligand that synergistically induces interleukin-6 in the presence of inflammatory signaling. Toxicol Sci. (2010) 115:89–97. 10.1093/toxsci/kfq024 20106948 PMC2855350

[60] Thomas SR Stocker R. Antioxidant activities and redox regulation of interferon-gamma-induced tryptophan metabolism in human monocytes and macrophages. Adv Exp Med Biol. (1999) 467:541–52. 10.1007/978-1-4615-4709-9_67 10721098

[61] Dounay AB Anderson M Bechle BM Campbell BM Claffey MM Evdokimov A. Discovery of brain-penetrant, irreversible kynurenine aminotransferase II inhibitors for schizophrenia. ACS Med Chem Lett. (2012) 3:187–92. 10.1021/ml200204m 24900455 PMC4025856

[62] Kozak R Campbell BM Strick CA Horner W Hoffmann WE Kiss T. Reduction of brain kynurenic acid improves cognitive function. J Neurosci. (2014) 34:10592–602. 10.1523/JNEUROSCI.1107-14.2014 25100593 PMC6802596

[63] Linderholm KR Alm MT Larsson MK Olsson SK Goiny M Hajos M. Inhibition of kynurenine aminotransferase II reduces activity of midbrain dopamine neurons. Neuropharmacology. (2016) 102:42–7. 10.1016/j.neuropharm.2015.10.028 26514401

[64] Parli CJ Krieter P Schmidt B. Metabolism of 6-chlorotryptophan to 4-chloro-3-hydroxyanthranilic acid: a potent inhibitor of 3-hydroxyanthranilic acid oxidase. Arch Biochem Biophys. (1980) 203:161–6. 10.1016/0003-9861(80)90164-2 6893263

[65] Yates JR Heyes MP Blight AR. 4-chloro-3-hydroxyanthranilate reduces local quinolinic acid synthesis, improves functional recovery, and preserves white matter after spinal cord injury. J Neurotrauma. (2006) 23:866–81. 10.1089/neu.2006.23.866 16774472

[66] Yates JR Gay EA Heyes MP Blight AR. Effects of methylprednisolone and 4-chloro-3-hydroxyanthranilic acid in experimental spinal cord injury in the guinea pig appear to be mediated by different and potentially complementary mechanisms. Spinal Cord. (2014) 52:662–6. 10.1038/sc.2014.118 25047053

[67] Platten M Ho PP Youssef S Fontoura P Garren H Hur EM. Treatment of autoimmune neuroinflammation with a synthetic tryptophan metabolite. Science. (2005) 310:850–5. 10.1126/science.1117634 16272121

[68] Yan Y Zhang GX Gran B Fallarino F Yu S Li H. IDO upregulates regulatory T cells via tryptophan catabolite and suppresses encephalitogenic T cell responses in experimental autoimmune encephalomyelitis. J Immunol. (2010) 185:5953–61. 10.4049/jimmunol.1001628 20944000 PMC2998795

[69] Lim CK Bilgin A Lovejoy DB Tan V Bustamante S Taylor BV. Kynurenine pathway metabolomics predicts and provides mechanistic insight into multiple sclerosis progression. Sci Rep. (2017) 7:41473. 10.1038/srep41473 28155867 PMC5290739

[70] Nourbakhsh B Bhargava P Tremlett H Hart J Graves J Waubant E. Altered tryptophan metabolism is associated with pediatric multiple sclerosis risk and course. Ann Clin Transl Neurol. (2018) 5:1211–21. 10.1002/acn3.637 30349856 PMC6186945

[71] Erhardt S Pocivavsek A Repici M Liu XC Imbeault S Maddison DC. Adaptive and behavioral changes in kynurenine 3-monooxygenase knockout mice: relevance to psychotic disorders. Biol Psychiatry. (2016) 82:756–65. 10.1016/j.biopsych.2016.12.011 28187857 PMC5812460

[72] Fallarino F Grohmann U You S McGrath BC Cavener DR Vacca C. The combined effects of tryptophan starvation and tryptophan catabolites down-regulate T cell receptor {zeta}-chain and induce a regulatory phenotype in naive T cells. J Immunol. (2006) 176:6752–61. 10.4049/jimmunol.176.11.6752 16709834

[73] Cronin SJF Seehus C Weidinger A Talbot S Reissig S Seifert M. The metabolite BH4 controls T cell proliferation in autoimmunity and cancer. Nature. (2018) 563:564–8. 10.1038/s41586-018-0701-2 30405245 PMC6438708

[74] Krause D Suh HS Tarassishin L Cui QL Durafourt BA Choi N. The tryptophan metabolite 3-hydroxyanthranilic acid plays anti-inflammatory and neuroprotective roles during inflammation: role of hemeoxygenase-1. Am J Pathol. (2011) 179:1360–72. 10.1016/j.ajpath.2011.05.048 21855684 PMC3157215

