# Supplementary material for: Co-treatments to Boost IDO Activity and Inhibit Production of Downstream Catabolites Induce Durable Suppression of Experimental Autoimmune Encephalomyelitis
Source: Front Immunol. 2020 Jun 17;11:1256. doi: 10.3389/fimmu.2020.01256 (PMC7311583; doi:10.3389/fimmu.2020.01256)
Supplement: Supplementary file 1 [file Data_Sheet_1.docx]

**Supplemental Figures:**

**Supplemental Figure S1.**

**
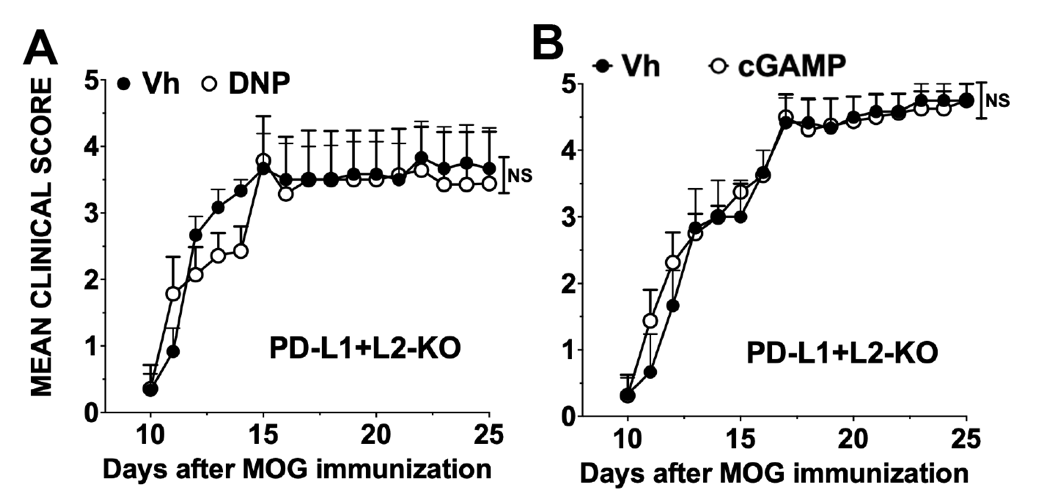
**

**Supplemental Figure S1. The PD-1/PD-L pathway is required for therapeutic responses to STING activators.** EAE was induced in PD-L1+L2-KO mice and disease onset and progression was monitored and scored. At EAE onset (day 11) mice were treated every other day with DNPs (A) or cGAMP (B) or vehicle (Vh, glucose 5% for A and saline for B) until day 21. Data were analysed by two-way ANOVA and are representative of 2 experiments with n=6-12. NS, not significant.

**
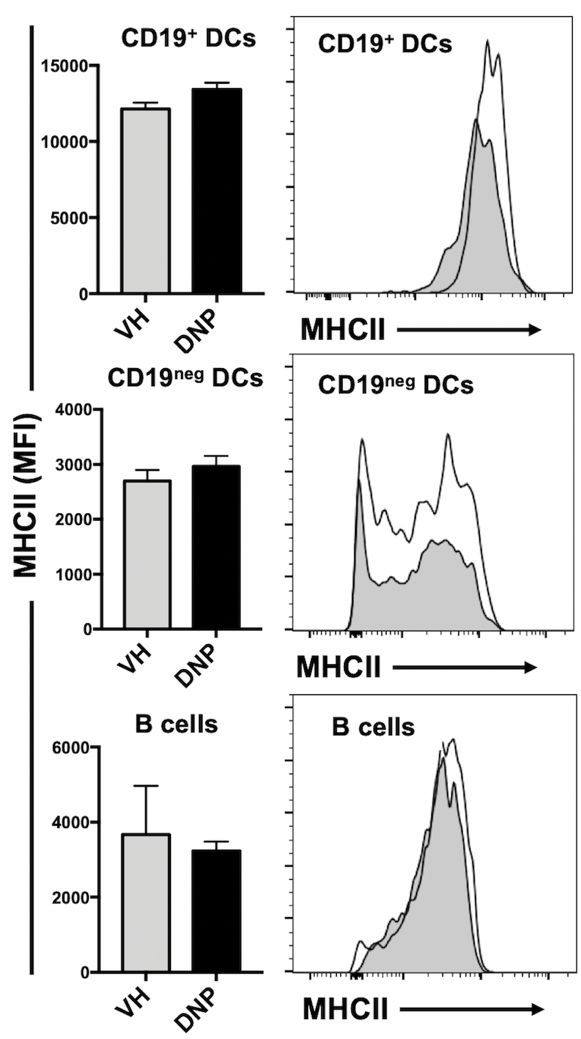
**

**Supplemental Figure S2. DNP treatment does not alter MHCII expression by splenic APCs.** EAE was induced in B6 (WT), and experimental groups were treated with either DNP or its vehicle (VH; 5% glucose) from day 11-21, every other day. On day 22 splenocytes were harvested and stained for CD11c, CD11b, CD19 and MHCII and analysed by flow cytometry. Data show mean MHCII expression levels for CD19^+^ and CD19^neg^ DCs and B cells (left) and a representative MHCII expression profile for each APC subset (right). Statistical significance was determined by two tailed unpaired Student’s *t* test; no significant variance in MHCII levels due to DNP treatments were detected for any APC subset.

**
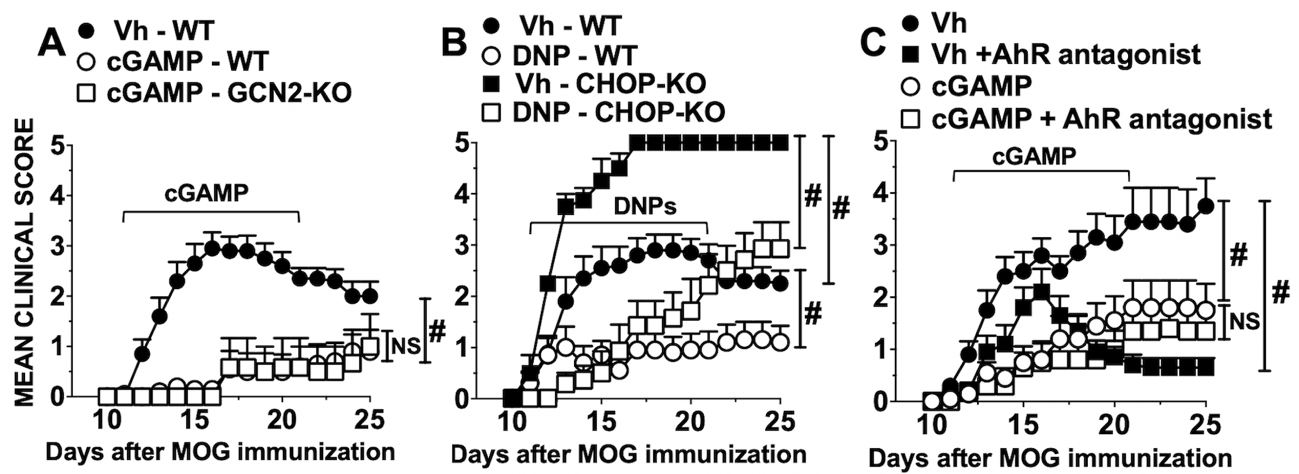
**

**Supplemental Figure S3. GCN2 or AhR signaling are dispensable for therapeutic responses to STING agonists. AB.** B6 (WT), GCN2-KO (A) and CHOP-KO (B) mice were treated with DNPs, cGAMP or Vh (glucose 5%) as depicted in figure 1A. Some mice were also treated with the AhR antagonist CH223191 (5 mg/kg of body weight, i/p., 1hr before each cGAMP injection). Data were analyzed by two-way ANOVA. Experiments were repeated once. ***p<0.001, ^#^p<0.0001.

**
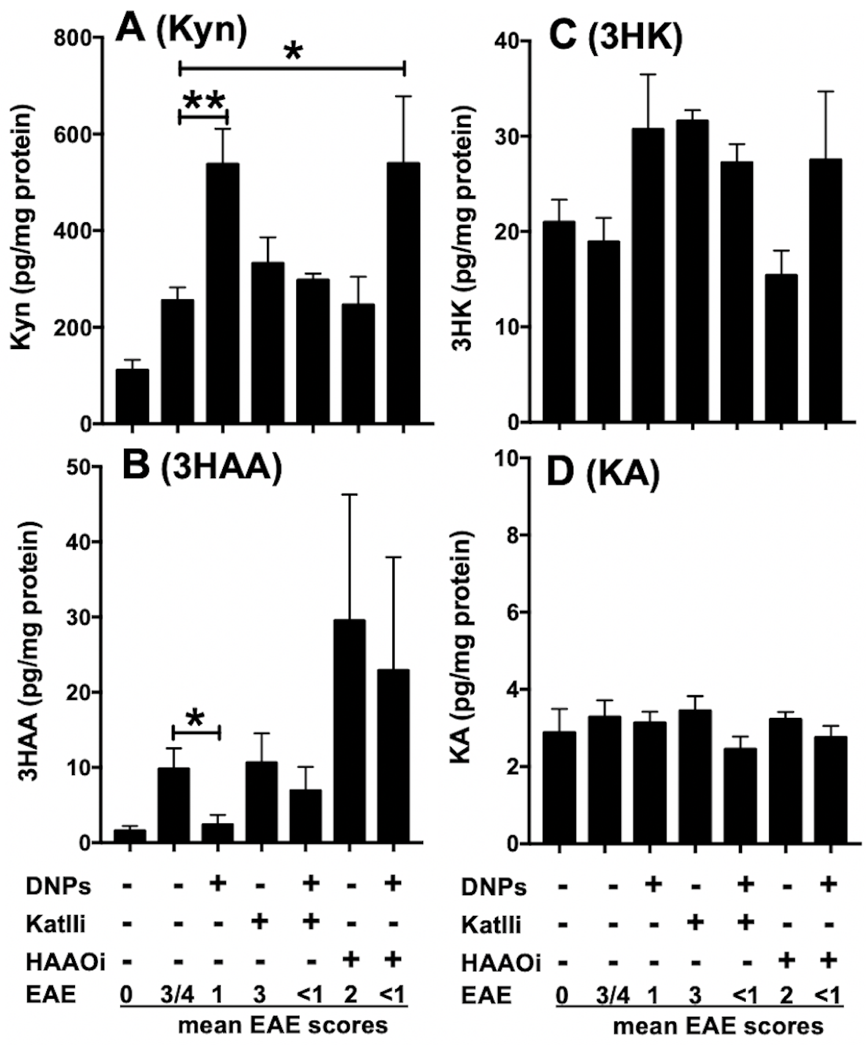
**

**Supplemental Figure S4. Metabolomic analysis of Trp catabolites.** EAE was induced in B6 mice (day 1) and at EAE onset mice were treated with DNPs with and without KatII (KatIIi) or HAAO inhibitors (HAAOi), as in Figure 6. Brain tissues were harvested on day 14. Brain tissues were homogenised and subjected to MS-LC/LC analysis to detect Kyn (A), 3HAA (B), 3HK (C) and KA (D) as described in Methods. **p<0.001, **p<0.001.
